# Recent Progress in Brain Network Models for Medical Applications: A Review

**DOI:** 10.34133/hds.0157

**Published:** 2024-07-08

**Authors:** Chenfei Ye, Yixuan Zhang, Chen Ran, Ting Ma

**Affiliations:** ^1^ International Research Institute for Artificial Intelligence, Harbin Institute of Technology at Shenzhen, Shenzhen, China.; ^2^ Department of Electronic and Information Engineering, Harbin Institute of Technology at Shenzhen, Shenzhen, China.; ^3^ Peng Cheng Laboratory, Shenzhen, China.; ^4^ Guangdong Provincial Key Laboratory of Aerospace Communication and Networking Technology, Harbin Institute of Technology at Shenzhen, China.

## Abstract

**Importance:** Pathological perturbations of the brain often spread via connectome to fundamentally alter functional consequences. By integrating multimodal neuroimaging data with mathematical neural mass modeling, brain network models (BNMs) enable to quantitatively characterize aberrant network dynamics underlying multiple neurological and psychiatric disorders. We delved into the advancements of BNM-based medical applications, discussed the prevalent challenges within this field, and provided possible solutions and future directions. **Highlights:** This paper reviewed the theoretical foundations and current medical applications of computational BNMs. Composed of neural mass models, the BNM framework allows to investigate large-scale brain dynamics behind brain diseases by linking the simulated functional signals to the empirical neurophysiological data, and has shown promise in exploring neuropathological mechanisms, elucidating therapeutic effects, and predicting disease outcome. Despite that several limitations existed, one promising trend of this research field is to precisely guide clinical neuromodulation treatment based on individual BNM simulation. **Conclusion:** BNM carries the potential to help understand the mechanism underlying how neuropathology affects brain network dynamics, further contributing to decision-making in clinical diagnosis and treatment. Several constraints must be addressed and surmounted to pave the way for its utilization in the clinic.

## Introduction

Connectome, the complete architecture of structural connectivity (SC) of the nervous system, plays a crucial part in orchestrating the interactions of neural activities across brain areas. A key goal of connectome-wide association study (CWAS) is to examine how alteration of brain networks may lead to neurological disorders [1]. Recent research has yielded a plethora of findings indicating that neurological conditions not only stem from localized brain damage but also could originate from widespread pathological alterations across the interconnected nervous system [2,3]. Take focal epilepsy as an instance, seizures that are synchronized emanate from a local region, known as the epileptogenic zone (EZ), expressing pathological discharges [4]. Such focal epileptiform discharges generated from EZ may propagate through large brain networks, affecting other healthy regions [5,6]. Advances in multimodal neuroimaging techniques, particularly descriptive analysis on empirical data, have progressively enabled the mapping of brain’s anatomical connectivity and the documentation of functional interactions between its various areas. As our comprehension of the biological underpinnings of brain diseases advances (focusing on molecular processes, genetics, and neuroimaging), there is a burgeoning interest in exploring how alterations in connectome contribute to the onset, development, and clinical trajectory of these disorders [7]. For example, a rising number of studies showed that neurodegenerative disorders could be characterized by the spread of neuropathological proteins (such as amyloid, tau, and α-synuclein) throughout brain connectome. Interestingly, the type of affected brain subnetwork may determine the specific symptomatic phenotype of the same neurological disorders (see reviews in [8]). This hypothesis was partly validated by connectomic neuromodulation, highlighting the key role of brain connectome in the symptom-specific treatments. Akram et al. [9,10] showed that surgical interventions involving deep brain stimulation (DBS) of the subthalamic nucleus (STN) for Parkinson’s disease (PD) can be predictive of symptom alleviation. SC between the DBS target and the supplementary motor area can predict enhancements in the symptoms of slow movement (bradykinesia) and stiffness (rigidity), whereas the connectivity to the primary motor cortex correlates with a reduction in tremor.

Given that spontaneous functional activity transmitted through human connectome accounts for producing a plethora of behavioral disturbance, there is a clear need to comprehend the fundamental mechanisms of brain activity as they are manifested through various neuroimaging techniques, such as electroencephalography (EEG), magnetoencephalography (MEG), and functional magnetic resonance imaging (fMRI), along both spatial and temporal dimensions [11]. As opposed to traditional descriptive approaches solely based on empirical connectome data (such as graph theory analysis), generative models of brain dynamics [12–14] emerge as a novel framework in the network neuroscience, by manipulating or perturbing networks in targeted and deliberate ways. During the preceding decade, multiple brain network models (BNMs) have been proposed to infer the internal states and dynamic processes of the brain cognitive function (see literature reviews in [4,15,16]) through mathematical representation of neural clusters in the form of neural populations or neural mean fields.

A large-scale BNM consists of a collection of coupled neural mass models (NMMs), which typically describe the neural activities of interacting brain regions, which further represent the desired neurophysiology or dynamic profile of the neural populations. The development of BNMs has primarily concentrated on the brain cortex [17–20], yet there is a growing trend to incorporate additional noncortical elements [21–24]. These regional NMMs of BNMs approximate the average ensemble behavior of neuron collections, rather than examining the intricate interplays among discrete neurons [15,17,25,26], which present a chance to quantify and elucidate the relationship between the structural attributes of neural circuits and the array of cerebral capabilities. The choice of the regional model often depends on striking a balance between model simplicity and realism. Recently, BNMs are deployed to explore aberrant network dynamics within the realm of neuropsychiatric conditions, encompassing Alzheimer’s disease (AD) [27–31], epilepsy [4,32–38], and schizophrenia [39,40], as well as stroke [41–43] and brain tumors [44,45]. Although several literature review papers have summarized the advantages and challenges of BNMs from the technical point of view [11,46,47], a comprehensive landscape of BNM-based medical applications in neuropsychological disease is still missing.

For this rapidly growing field, this paper intends to review the advanced BNMs with their representative medical applications. First, we give a brief overview of some classic BNM approaches and the related software. Then, several BNM medical applications in various brain diseases are presented. Last, we address the current challenges in this field and provide possible solutions for future research.

## Brain Network Models

BNMs enable us to quantitatively characterize spatiotemporal dynamics of the brain functional activity in a multiscale view. The architecture of a BNM can be viewed as a graph composed of nodes (NMMs) linked through edges (connectivity), expressing higher-order interactions. Brain network nodes could correspond to either clusters of neurons or distinct brain areas, which is accomplished by segmenting the entire cerebral cortex into numerous minute regions. The edges of the graph denote either SC or functional connectivity (FC) obtained from multimodal neuroimaging data. Research indicates a strong correlation between SC and FC, whereas the one-to-one correspondences between them are constrained. The interconnected structure of the human cerebrum inherently obscures the correspondence of structure and function [48]. Considering the poor direct correlation between SC and FC, NMMs are a set of stochastic differential equations constructed to express higher-order interactions between brain regions, establishing a mapping from structural to functional networks.

**Fig. 1.**
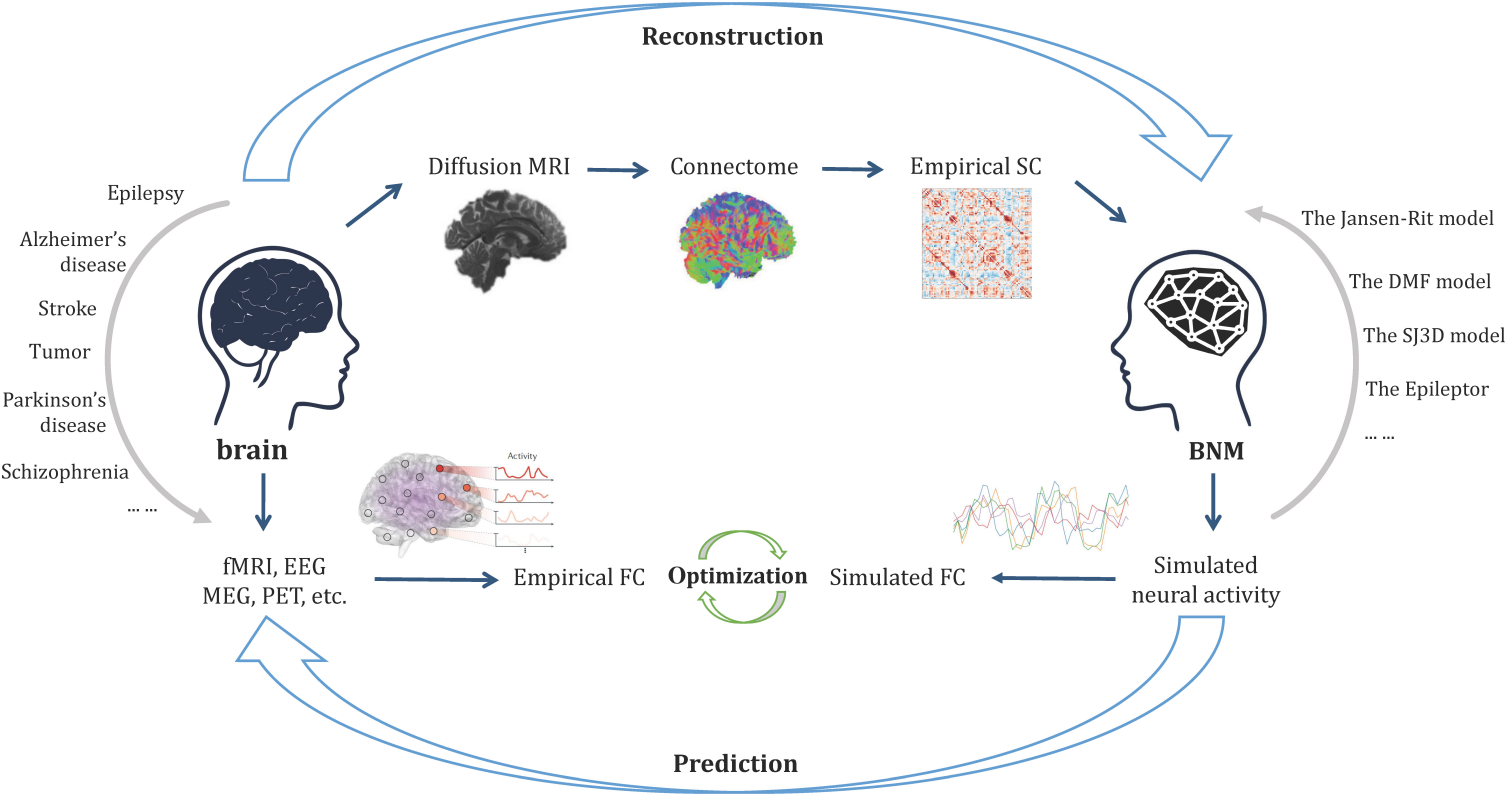
A disease-oriented BNM pipeline. Diffusion MRI data are processed to obtain individual large-scale connectome with empirical brain SC (top row). Empirical FC is extracted through statistical analyses of temporal data from MEG, EEG, or fMRI (bottom left). The BNM is constructed by coupling NMMs with SC data and supplies detailed computational neuronal dynamics for each brain area (bottom right). Then, output signals of model simulation are compared against empirical functional data to estimate the optimal working points and regimes of a virtual brain (bottom center).

Brain modeling approaches allow to detect individual variation across patients with specific diseases. Recent investigations have revealed that fitting the neural output from neurodynamic simulations to personalized fMRI scans can derive individual-specific parameter estimates that are associated with pertinent clinical behavioral measurement [27,49]. For computational medical applications, brain network modeling enables the individualized perturbation of parameters that reflect the alterations associated with particular neurological conditions [50]. Thus, BNMs may provide guidance for hypothesizing changes in neurophysiological states of individual subjects and characterize neurodynamics in specific disorders [51]. A general disease-oriented BNM pipeline is illustrated in Fig. 1. Briefly, individual brain anatomy and large-scale connectome (SC) can be obtained from structural and diffusion tensor imaging (DTI) data preprocessing. Empirical FC is extracted through statistical analyses of temporal data acquired via MEG, EEG, or fMRI. The global BNM is then constructed by coupling local NMMs through SC data and can help simulate large-scale brain activity. For optimization, simulated signals are fitted to empirical data to get the optimal working points (i.e., the parameters of the model that best fit the empirical data, see Table 1 for more explanation) and regimes of a BNM.

**Table 1.** Glossary of terms and abbreviations

Terms	Interpretations
Connectome	A comprehensive atlas of neurological connectivity in the brain.
Structural connectivity (SC)	Anatomical connections between neurons or brain regions. Such as axonal or synaptic connections between neurons, and nerve fiber orientation distribution in the cortex and subcortical nuclei.
Functional connectivity (FC)	The strength of the connection between 2 brain ROIs, usually determined by calculating the relationship between regional neurophysiological signals using correlations or mutual information.
Neural mass models (NMMs)	A model describes neural function at the mesoscopic level, versus the single neuron models. NMMs are able to exactly predict recorded signals of brain areas (e.g., EEG, MEG, and fMRI BOLD signals), describing the coherent activity of entire populations of neurons.
Resting state (RS)	The state in which humans are awake, eyes closed, and relaxed.
Neuromodulation	Modulation via ascending projections from the brainstem and subcortical nuclei alters neuronal firing rate, making neurons more or less responsive to incoming signals. By adjusting neural gain, the nervous system can achieve multiple types of functional connectivity patterns on the same underlying structural network.
Working point	The model parameters that allow the simulation output to best fit the empirical data.
Participation coefficient	The connection strength of a brain node within its brain network.
Higher-order interactions	Refers to the state variable representing a node is influenced by a nonlinear combination of the states of several other nodes in a network. Higher-order interactions determined by the network topology can reflect the complex mapping relationships between structural and functional connectivity.

BOLD, blood oxygen level dependent; EEG, electroencephalogram; fMRI, functional magnetic resonance imaging; MEG, magnetoencephalogram; ROIs, regions of interest

In this section, we aim to introduce several popular BNMs in current researches.

### The Jansen–Rit model

Early NMMs emerged from meticulous scrutiny of the aggregate reactions within a group of neurons to changes in driving inputs. The Wilson and Cowan model [17] comprises 2 neural populations (i.e., one excitatory and one inhibitory population), serving as a simplified depiction of an NMM. This backbone model has been developed to more complicated extensions [20,52]. For instance, the proposed Jansen and Rit model [15] consists of 3 neural masses: a pair of excitatory and a group of inhibitory interneurons, which demonstrate more biological plausibility. Here, we mainly focus on the Jansen–Rit model.

The Jansen–Rit model [15] features a fundamental network of an elementary circuit of 3 interacting neural clusters (symbolizing assorted cellular assemblies) that delineate a brain cortical area (or column): pyramidal cells, inhibitory interneurons, and excitatory interneurons. This model represents a biologically motivated mathematical representation initially designed to reproduce the inherent electrical dynamics within neural clusters, highlighting the alpha rhythm specifically [15]. The dynamic behaviors of the Jansen–Rit model is intricately tied to its parameters [53], illustrating the interplay among various neural populations (by parameters of long-distance coupling and local interaction) and delineating the specific neural masses (excitatory and inhibitory) that receive external noise, stimuli, and inputs of thalamic nuclei (by parameter of input strength). For instance, alterations in the dynamics of excitatory and inhibitory postsynaptic membrane potentials (PSPs) (e.g., modifications in the temporal parameters of the mathematical framework) will affect the model behavior (e.g., accelerating or decelerating local dynamics). In a specific case, the proportion of excitatory and inhibitory time constants of the Jansen–Rit model was computationally estimated for measuring the correlation between amyloid-β (Aβ)-related synaptic disinhibition and the EEG slowing characteristic of AD [28]. Additionally, the Jansen–Rit model has been investigated extensively, enabling the simulation of physiological signals detected through various recording methods such as intracranial local field potentials (LFPs), stereo-EEG (sEEG), scalp EEG, and MEG. For example, it generated reactions similar to those observed in evoked potentials (EPs) following a series of impulses [25], oscillations in the high-alpha and low-beta range upon the incorporation of recurrent inhibitory connection and spike-rate modulation within the inhibitory model [54], and seizure patterns to emulate temporal lobe epilepsy [55].

### The dynamic mean field model

This NMM of cerebral cortex was initially introduced by Wong and Wang [56], which is a dual-variable model and can be visualized as an interconnected network of neuronal populations regulated by mutual inhibition [56]. Deco [16] further simplified the Wong–Wang model into a single neuronal framework. The dynamic mean field (DMF) model serves as an estimation for a spiking network model [16,57], which encompasses populations of both excitatory and inhibitory neurons, representing the collective activity of each population through a single variable of the average firing rate. Within the perspective of DMF approach, the resultant firing rate for each group of neurons is predicated on the currents they receive, which are reciprocally shaped by the firing rates. As a result, the firing rates across neural populations are calculated in a self-consistent manner through a simplified set of coupled nonlinear differential equations [58].

The local excitation-inhibition (E–I) ratio is determined by the excitatory connection strength and the adjustable local inhibitory feedback synaptic strength parameters of the DMF model (see Table 2), which has important implications for spontaneous spiking activity. Controlling the local feedback inhibitory parameter can enhance the information transfer capacity (i.e., mapping different network inputs into distinguishable outputs) at the whole-brain level [59]. The DMF modeling methods are suitable for characterizing local cerebral E–I physiology changes and for exploring the impact of the cortical circuitry’s physiological state on FC [50]. Modulating the local E–I ratio of the model allows for investigating how neurodegenerative changes affect large-scale brain dynamics and cognitive function. Each cortical region modeled with the DMF model can be coupled in accordance with the personalized empirical structural connectome and weighted by multiplying with global scaling coefficient further to replicate expansive cerebral dynamics and facilitate research into the FC of brain networks [44,60]. The DMF model has been adopted in investigating brain dynamics for several neuropsychological disorders, including AD [27,30], schizophrenia [39], and stroke [58,61].

**Table 2.** Basic elements of neural mass models

Neural mass model	Key parameter	Description
Jansen–Rit model	τ_e_	Excitatory dendritic time constant
	τ_i_	Inhibitory dendritic time constant
	v_0_	Mean PSP threshold for 50% of maximum firing rate
	C_1_	Average number of synaptic contacts from excitatory to pyramidal cells
	C_2_	Average number of synaptic contacts from pyramidal to excitatory cells
	C_3_	Average number of synaptic contacts from inhibitory to pyramidal cells
	C_4_	Average number of synaptic contacts from pyramidal to inhibitory cells
	m_3T,0_	Input firing rate at the pyramidal cells
Dynamic mean field (DMF) model	I_syn_	Total synaptic input current
	J_NMDA_	Strength of excitatory (NMDA) synaptic coupling parameter
	J_i_	Strength of the local feedback inhibitory (GABA) synaptic coupling for each area *i*
	w_+_	Strength of local excitatory recurrence parameter
Stefanescu–Jirsa 3D (SJ3D) model	K_11_	Connectivity strength between neurons within the excitatory subpopulation
	K_12_	Connectivity strength between excitatory and inhibitory neurons
	K_21_	Connectivity strength between inhibitory and excitatory neurons
Epileptor	z	Slow permittivity variable describing the systemic effects that dictate how close the system is to the seizure threshold
	(x_1_, y_1_)	First oscillator subsystem describing the fast discharges
	(x_2_, y_2_)	Second oscillator subsystem involved in spike wave events
	a	Local Hopf bifurcation parameter
	x_0_	Degree of epileptogenicity of a brain region

GABA, GABA receptor; NMDA, NMDA receptor; PSP, postsynaptic potential

### The Stefanescu–Jirsa 3D model

Considering the inability of traditional approaches based on mean field theory to address synchronized neural activity, Assisi [62] defined a simplified representation to approximate the collective activities of brain, in particular different types of asynchronous behavior. Further, they extended the method to a biologically more realistic network, and then the Stefanescu–Jirsa 3-dimensional (SJ3D) model [26] was proposed. This model includes mixed excitatory and inhibitory networks focusing on field potentials, which can display spiking and bursting behavior. Originally extracted from coupled Hindmars–Rose neurons [63], these models are designed to generate both stimulating and fluctuating behaviors. The SJ3D model anticipates regional cerebral dynamics through a suite of 6 differential equations and includes parameters representing different physiological characteristics, such as neuron membrane potentials, the transmembrane movement of ions via rapid and sluggish ion channels, and the interactive linkage between populations of excitatory and inhibitory neurons. It is capable not only of accurately reproducing the mean field amplitude of the primary networks but also of seizing the pivotal temporal features of network fluctuations. Through this method, intricate dynamical events like multiclustered oscillations, synchronization across varying timescales as well as the cessation of oscillations can be effectively simulated within large-scale neural networks while incurring minimal computational expense.

The SJ3D model embedded in BNM framework has been implemented in understanding neural rehabilitation mechanism. For example, it has been used to explain the functional mechanisms of stroke recovery [41,42]. Given the low dependence of the SJ3D model on synaptic delay, this feature makes it compatible with the low temporal precision inherent in the blood oxygenation level-dependent (BOLD) response of fMRI.

### The Epileptor

The Epileptor, first proposed by Jirsa [4], is a phenomenological model of neural populations for reproducing epileptic temporal seizures. This model is developed from a combination of nonlinear differential equations with 5 state variables, containing a fast timescale 2D subsystem, an intermediate timescale 2D subsystem, and a slow dielectric constant variable [4,64]. The Epileptor enables us to simulate the temporal separation of focal epileptic seizures and capture the progression and trajectories of seizures within the framework of state space.

Over the past decade, increasing simulation studies were carried out by applying the Epileptor or its derived models to investigate aberrant neural signal propagation in epilepsy [6,38]. For instance, Jirsa [65] proposed a novel personalized strategy toward epilepsy intervention by developing a Virtual Epileptic Patient (VEP) brain model, namely, a Epileptor-coupled individual model. Evidence showed that the spatiotemporal properties of seizure propagation could have been reliably estimated through this model [6]. Besides, by extending the Epileptor into 2 subtypes of neuron groups (i.e., epileptogenic and nonepileptogenic), Courtiol [35] unraveled new dynamical mechanisms underlying altered FC of epileptic brains under the resting state through generating natural brain rhythms alongside the seizure-associated patterns [35].

## Implementation Software

A cornerstone of BNM is computational simulation based on high-dimensional neurophysiological data. Thus, robust software for modeling and emulating human brain is essential to the BNM research community. Among this section, we will briefly describe several software designed to simulate collective whole-brain dynamics, as well as their workflow for the implementation of BNMs (see Table 3 for details).

**Table 3.** BNM implementation software

Software framework	URLs	Equipped with GUI or not	Programming language	NMMs
The Virtual Brain (66,67,70,71)	https://www.thevirtualbrain.org/	Yes	Python, MATLAB, C	Generic 2D oscillator, the Wilson–Cowan model, the Jansen and Rit model, the dynamic mean field model, the Stefanescu and Jirsa 3D model, the Epileptor, etc.
neurolib (72)	https://neurolib-dev.github.io	No	Python	The Hopf model, the ALN model, the Wong–Wang model, the Wilson–Cowan model, the Fitz–Hugh Nagumo model, etc.
NEST (73,74)	https://www.nest-initiative.org/	No	Python	The integrate and fire (IAF) neuron model, the MAT2 neuron model, the Hodgkin–Huxley neuron model, simple multicompartment neuron models, the mean field model, etc.
Brain (75)	https://github.com/brian-team/	No	Python	The Hodgkin–Huxley model, the Wang–Buszaki model, the Jansen and Rit model, the adaptive exponential integrate and fire (IAF) model, etc.
GENESIS (76)	http://genesis-sim.org/	Yes	Python	The phase equation model of coupled oscillators, the multicellular model of the mammalian olfactory cortex, the model of orientation selectivity in the visual system, etc.

GUI, graphical user interface; NMM, neural mass model

A general pipeline to simulate global brain network dynamics takes biologically plausible connectivity derived from fiber-tracking datasets, including DTI and diffusion spectrum imaging (DSI), to set the intensity of connectivity and propagation lags across all network nodes via signal transmission. To define the dynamics of a brain region, numerous NMMs can be configured within or uploaded to the software. Both the NMMs and SC define the whole-brain network model. In a simulation, each brain area generates a range of neural signals encompassing LFPs and neuronal firing rate, alongside neuroimaging measurements like EEG, MEG, and the BOLD activity scanned by fMRI. To optimize an individual model, the pipeline takes parameter exploration operations to characterize simulated behavior as a function of changing parameters such as the bifurcation parameter (representing the excitability of a brain area) and coupling parameter (representing the coupling strength among disparate areas of the brain). Researchers often analyze the simulated output of a BNM in comparison with subject-specific empirical fMRI recordings.

### The Virtual Brain

The Virtual Brain (TVB; thevirtualbrain.org) offers an open-source neuroinformatics platform [66,67] that facilitates the development, simulation, and analysis of whole-brain network models for macroscopic brain simulations. TVB offers a wide range of NMMs as regional models of a large-scale BNM. Manipulations of network parameters (e.g., the epileptogenicity and excitability parameters in an epileptic brain model) within TVB empowers scientific investigators and clinicians to assess the impacts of research methodologies, interventions (e.g., brain stimulation and surgical procedures), and therapeutic approaches (e.g., drug therapies aimed at specific brain regions) [68]. The computational environment framework allows users to visualize the simulation outcomes in both 2D and 3D formats, as well as to conduct analytical procedures similar to those typically performed within empirical data.

TVB is readily accessible via the web GUI of EBRAINS, and it also functions as a Python library for developing scripts within the EBRAINS Lab environment. Through these interfaces, users are able to upload models of brain networks, adjust settings, initiate simulations, and subsequently postprocess and analyze outcomes. There are multiple NMMs already integrated within TVB. The implementation facilitates a methodical investigation and adjustment of all intrinsic elements within a large-scale BNM [69], including the NMMs that direct regional fluctuations or the structural connection that dictates the spatiotemporal framework of the interconnected network. The foundational approaches of TVB software are extensively described among multiple publications [66,67,70,71] as well as in the online documentation (docs.thevirtualbrain.org).

### Others

There are other BNM software especially beneficial for modeling extensive brain networks and neural dynamics, such as neurolib [72], NEST [73,74], Brain [75], and GENESIS [76]. These software frameworks are ideal to help user streamline the development, simulate large networks, analyze output signals, and optimize the biological neuronal models. Among the frameworks mentioned above, neurolib [7*2*] is a free open-source Python framework designed for large-scale brain modeling. For model construction, users can choose from different NMMs implemented in neurolib or their own NMMs to reproduce the dynamics within individual brain region. neurolib helps researchers to load and handle structural and functional brain data, to analyze brain simulations, as well as to tune model’s parameters and fit it to empirical data. In addition, NetPyNE [77] and NEURON [78] are particularly suited to simulate large networks of spiking neurons. Nengo [79] and Brain Modeling Toolkit [80] were specifically designed for simulating mesoscopic neural systems, which are not optimized for large-scale cerebral simulation primarily because of the extensive computational expenses in calculating large amount of parameters.

## Medical Applications

**Fig. 2.**
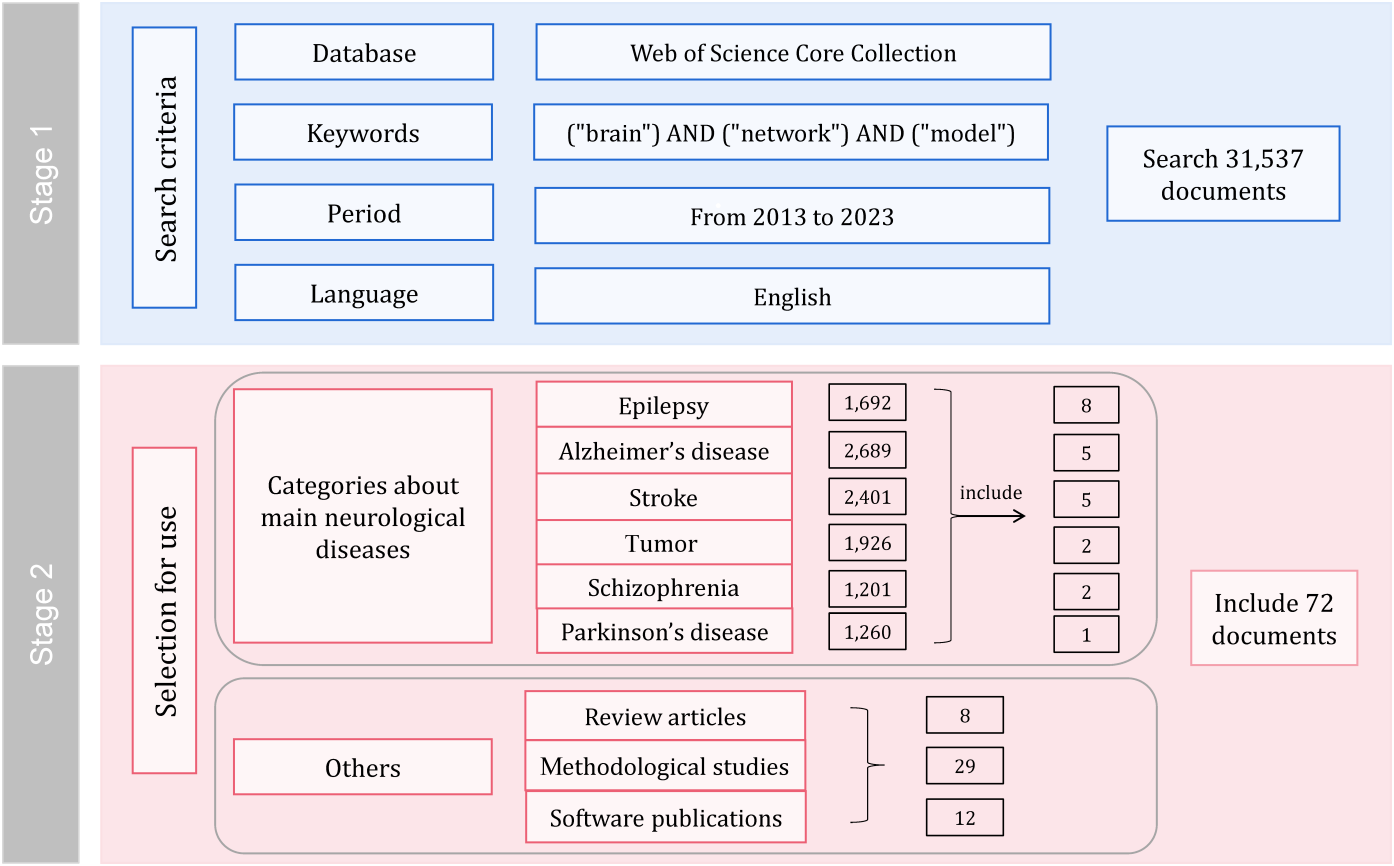
Literature selection flowchart.

Alternative to the traditional descriptive brain analytical models, which are solely based on empirical neurophysiological data, the generative BNMs enable to simulate a data-driven “digital twin” of the subject’s brain [49]. In other words, a personalized BNM generated from individual anatomical connectivity, optimized by fitting to functional neuroimaging datasets, would facilitate the examination of clinical hypotheses and the investigation of innovative treatment methodologies for target subject. This paradigm shift manifests the advantage to perturb and characterize individual brain dynamic in neurological diseases, thus opening up new avenues toward deepening our understanding of disease mechanism. We therefore performed a comprehensive review of recent literature to assess the landscape of BNM medical applications (as shown in Fig. 2).

For the literature selection, we searched *Web of Science Core Collection* from 2013 to December 2023, using keywords including “brain,” “network,” and “model”. According to the initial search findings of the first step, we added the constraint terms including “epilepsy,” “Alzheimer’s disease,” “stroke,” “tumor,” “schizophrenia,” and “Parkinson’s disease,” respectively, to obtain literatures regarding BNM applications on brain diseases. The language of the literature was restricted to English. All article titles and abstracts of the retrieved papers were screened, and the complete content of those potentially relevant works was accessed and obtained. The bibliographies of selected studies were further examined to uncover supplementary research. Subsequently, we performed the search and review all articles. We included studies if they were published as full text articles and used BNM to give the pathological or interventional mechanisms, as well as predictive and prognostic for several brain diseases mentioned above. Furthermore, we filtered the search results from the first step and included review articles of BNMs, methodological studies on several classical NMMs, and publications of modeling software. Duplicate publications were excluded.

The final 23 selected literatures were listed in Table 4. We will summarize the BNM medical applications as below.

**Table 4.** Literature of BNM medical applications

Type of Disease	Index	Reference	NMM	Sample size	Data type	Atlas	Software	Model metrics	Findings
Epilepsy	1	Jirsa et al. (65)	Epileptor	15 epileptic patients, 5 healthy controls	sEEG, sMRI, dMRI	Desikan–Killiany Atlas	TVB	The Jacobian matrix in linear stability analysis on the 2D Epileptor model	(1) A large extent of the estimated propagation zone is not explored by sEEG and is significantly correlated with poor seizure prognosis according to the Engel classification; (2) The topology of the connectivity matrix for BNM is significantly important to predict the recruited network; (3) Weak coupling is a crucial assumption for the prediction of recruited networks.
	2	Proix et al. (33)	Epileptor (coupled to the VEP model)	One epileptic patient	sEEG, sMRI, dMRI	Parcellation template used for whole brain tractography	TVB	Partial distribution of excitability parameter *x_0_* of Epileptor	(1) Changes of excitability in the EZ regions show fairly little influence on the number of seizures in the VEP brain model, whereas reduction of excitability outside of EZ/PZ regions is linked to seizure reduction in the left thalamus and hypothalamus, and to a lesser extent in the left parahippocampus; (2) With posterior distribution of the model parameters given the data, the propagation of the seizure between areas can be correctly reconstructed and informative estimates can be obtained for EZ; (3) The VEP approach provides a non-invasive approach toward the evaluation of the best placement of the sEEG electrodes; thus, the EZ hypotheses are improved and surgical strategies can be systematically tested within the VEP model.
	3	Proix et al. (34)	Epileptor neural field model	13 epileptic patients	sEEG, dMRI	Desikan–Killiany Atlas	TVB	(1) Emergence of low-voltage fast activity (LVFA) oscillations, (2) ictal SWDs	(1) The ictal wavefront acts as the main “generator” of ictal activity; when it dies out, the seizure terminates synchronously; (2) If one of the offset bifurcations propagates much faster than the ictal wavefront, involving the full extent of the recruited neural field, then epilepsy seizures in which the source of SWDs remains stationary.
	4	Olmi et al. (6)	Epileptor	15 epileptic patients	PET, EEG, sEEG, dMRI	Desikan–Killiany Atlas	TVB	Largest eigenvector related to the maximal (positive) eigenvalue of the Jacobian matrix *J* calculated in the steady state solution of Epileptor	(1) Patient-specific network connectivity is predictive for subsequent seizure propagation pattern; (2) Seizure propagation is characterized by a systematic sequence of brain states; (3) Seizure propagation can be controlled by an optimal intervention on the connectivity matrix; (4) The degree of invasiveness can be significantly reduced via the proposed seizure control as compared to traditional resective surgery; (5) Stability analysis of the network dynamics employing structural and dynamical information can estimate reliably the spatiotemporal properties of seizure propagation.
	5	Courtiol et al. (35)	Extended Epileptor	14 epileptic patients, 5 healthy controls	dMRI, rs-fMRI	Desikan–Killiany Atlas	TVB	①Local excitability parameter *a* of NMM, ②global coupling parameter *K_rs_* of the connectivity weights for Hopf subpopulations	(1) Epileptic brains during interictal resting state are associated with lower global excitability induced by a shift in the model working point, indicating that epileptic brains operate closer to a stable equilibrium point than healthy brains; (2) Functional networks are unaffected by interictal spikes; (3) Perturbations of local brain dynamics in epileptogenic brain regions trigger network-wide connectivity; (4) The dynamical mechanisms responsible for altered resting-state FC in epilepsy involving 2 key factors: (a) a shift of excitability of the whole brain leading to increased stability and (b) a locally increased excitability in the epileptogenic regions supporting the mixture of hyperconnectivity and hypoconnectivity in these areas.
	6	Hashemi et al. (36)	Epileptor	2 epileptic patients	EEG, sEEG, MEG, fMRI, dMRI	Desikan–Killiany Atlas	TVB for BNM simulations, PPL tools (Stan&PyMC3) for inverting the BNM	Spatial distribution of excitability parameters *a* for different brain nodes	(1) The proposed Bayesian VEP framework allows to infer the spatial map of epileptogenicity in a personalized large-scale brain model of epilepsy spread; (2) No-U-Turn Sampler (NUTS) and Automatic Differentiation Variational Inference (ADVI) algorithms can accurately estimate the degree of epileptogenicity of brain regions.
	7	Dollomaja et al. (38)	6D Epileptor model	One epileptic patient	sEEG, T1w, DWI	VEP atlas	TVB	Excitability *x_0_*	(1) Status epilepticus propagates in the brain under the constraint of the structural connectome, following the strongest estimated white matter connections; (2) Both the empirical and the simulated propagation patterns involve the regions connected to 2 seizure onset areas of the patient, and the 2 onset regions are highly connected to each other.
	8	Jirsa et al. (49)	Epileptor (coupled to the VEP model)	One epileptic patient	EEG, sEEG, fMRI, dMRI	VEP atlas	TVB	Simulated SEEG	(1) VEP simulation can help predict the extent of the EZ, the behavior of the non-operated regions, and the potential effectiveness of surgery, further improving postsurgical outcomes; (2) Additional information, such as previous knowledge of interictal activity or lesions observed on MRI, can be formally integrated in the inference process and reduce the uncertainty with VEP; (3) Some unresected epileptogenic regions identified by VEP were not sampled by the sEEG electrodes.
Alzheimer’s Disease (AD)	9	Zimmermann et al. (27)	Reduced Wong Wang model	16 AD patients, 35 aMCI participants, 73 healthy controls	sMRI, dMRI, rs-fMRI	AAL template	TVB	Model parameters: ①global coupling *G*, ②inhibition-excitation *L_IE_*, ③excitation-inhibition *L_EI_*, ④excitation-excitation *L_EE_*.	(1) Neuropsychological cognitive domain scores corresponded tightly with clinical diagnosis groupings; scores decreased consistently and significantly across the 3 clinical diagnosis groups (HC > MCI > AD); (2) The discrepancy between model parameters of the Limbic SubNet and the Whole Network correlates with cognitive performance. Individuals with higher cognitive scores (i.e., healthy subjects) have a greater discrepancy in inhibition and lower discrepancy in excitatory and global coupling parameters; (3) Large variance in the optimal values of excitation/inhibition and global dynamics exists among the MCIs compared to healthy controls and AD groups; (4) The effects of excitation and inhibition are often unpredictable for AD function.
	10	Stefanovski et al. (28)	Jansen–Rit model	10 AD patients, 8 MCI participants, 15 healthy controls	EEG, PET, DWI	HCP Multimodal Parcellation (92)	TVB	Postsynaptic membrane potential (PSP)	(1) Individual Aβ burden modulates regional Excitation–inhibition balance, leading to local hyperexcitation with high Aβ loads; (2) Neural slowing propagates to central parts of the network independently of the spatial Aβ distribution; (3) Virtual therapy with the NMDA antagonist memantine can lead to functional reversibility.
	11	Tait et al. (31)	Theta model (a phase oscillator model)	21 AD patients, 26 healthy controls	EEG, eLORETA	Brainnetome atlas	In-house code	Excitability *I_0_* of ROIS	(1) The most important ROIs for seizure generation were those typically burdened by Aβ at the early stages of AD; (2) Alterations to the large scale network structure in AD potentially play a role in determining seizure phenotype, namely, an increased likelihood of generalized seizures in AD.
	12	Nifterick et al. (29)	NMM to mimick EEG/MEG [Lopes da Silva et al. (21)]	18 SCD participants, 18 aMCI participants	MEG, PET, DTI	AAL template	In-house code	Average membrane potential of the main pyramidal neuronal populations	Alzheimer’s disease-mediated neuronal hyperactivity can lead to oscillatory slowing, likely due to (1) hyperexcitation (by hyperexcitability of pyramidal neurons or greater long-range excitatory coupling) and/or (2) disinhibition (by reduced excitability of inhibitory interneurons or weaker local inhibitory coupling strength) in early AD.
	13	Monteverdi et al. (30)	Wong–Wang neural mass model	16 AD patients, 7 FTD patients, 10 healthy controls	DWI, fMRI	(1) * ad hoc* atlas, (2) Buckner and Yeo cerebral and cerebellar functional atlases	TVB	(1) a connections strength parameter: the global coupling (*G*), (2) 3 synaptic parameters: the excitatory (NMDA) synapses (*J_NMDA_*), the inhibitory (GABA) synapses (*J_i_*), the recurrent excitation (*w_+_*)	(1) Model parameters markedly differentiated the mechanisms underlying brain networks dynamics in AD and FTD, with the most typical changes being concentrated in the DMN and LN of AD and in the FPN of FTD: (a) In AD and FTD pathologies, global coupling increased in DMN and decreased in LN, while it decreased in FPN in FTD only; (b) Synaptic parameters can differentiate AD from FTD; (c) Both in AD and FTD, the SMN showed reduced excitatory coupling (*J_NMDA_*) and increased recurrent excitation (*w_+_*). (2) Global coupling and synaptic parameters of both AD and FTD network significantly contributed to explain neuropsychological scores in specific cognitive domains. (3) Toward personalized fingerprints of AD and FTD patients, the most meaningful model biomarkers for patient’s labeling were *G* in DMN, *G* in LN, *J_i_* in AN.
stroke	14	Falcon et al., 2015 (41)	Stefanescu–Jirsa 3D (SJ3D) model	20 stroke patients, 10 healthy controls	T1w, DTI, rs-fMRI	Lausanne 2008 atlas	TVB	(1) global parameters: conduction velocity and long-range coupling; (2) local parameters: coupling between excitatory and inhibitory populations: *K_11_* (excitatory on excitatory), *K_12_* (excitatory on inhibitory), and *K_21_* (inhibitory on excitatory)	(1) Long-range model coupling is negatively correlated with global efficiency; the larger influence of local dynamics seen through the long-range coupling parameter is closely associated with a decreased efficiency of the system; (2) The increase in the long-range model parameter (indicating a bias toward local over global dynamics) is deleterious because it reduces communication as suggested by the decrease in efficiency.
	15	Adhikari et al., 2015 (58)	the Dynamic Mean Field (DMF) model	10 child patients, 31 age-matched control children	T1w, DSI, fMRI	(1) Desikan–Killiany Atlas, (2) the 1000-ROIs template defined by Cammoun et al. (98)	TVB	the global scaling factor *G* of each brain network	(1) The structural damage caused by an early brain injury is unlikely to have an adverse and sustained impact on the functional connections, albeit during the resting state, of damaged areas; (2) For children sustained stroke, the damaged areas could continue to play a role in the development of near-normal function in certain domains such as language.
	16	Falcon et al. (42)	the -Jirsa 3D (SJ3D) model	20 stroke patients, 11 age-matched healthy controls	T1w, DTI, fMRI	a parcellation template to transform the original macaque parcellation template (99) to the human MNI template (100)	TVB	(1) global parameters: conduction velocity and global coupling; (2) local parameters coupling between excitatory and inhibitory populations: *K_11_* (excitatory over excitatory), *K_12_* (excitatory over inhibitory), and *K_21_* (inhibitory over excitatory)	(1) Individuals with stroke demonstrated a consistent reduction in conduction velocity, increased local dynamics, and reduced local inhibitory coupling; (2) There is a negative relationship between local excitation and motor recovery, and a positive correlation between local dynamics and motor recovery.
	17	Adhikari et al. (61)	Dynamic mean field (DMF) model	36 stroke patients, 26 age-matched healthy controls	dMRI, fMRI	(1) Desikan–Killiany Atlas, (2) the 1000-ROIs template defined by Cammoun et al. (98)	In-house code	Global scaling factor *G* of the DMF model	(1) The integration and information capacity were decreased in stroke patients, as compared to healthy controls, particularly at the level of resting-state networks; (2) Focal lesions affect the brain’s ability to represent stimuli and task states, and that information capacity measured through whole brain models is a theory-driven measure of processing capacity that could be used as a biomarker of injury for outcome prediction or target for rehabilitation intervention.
	18	Idesis et al. (43)	Hopf computational model	96 stroke patients, 27 healthy controls	dMRI, fMRI	HCP-842 atlas	In-house code	Effective connectivity	(1) The critical importance of white matter damage not only for understanding the physiological effects of stroke but also for accurate modeling and prediction; (2) Functional alterations of brain networks are important for cognitive functions that rely on distributed networks (e.g., memory, attention, and language), as compared to visual and motor functions for which structural damage is more sensitive; (3) The DMN is the network that exerts the main influence over other networks and that this influence is significantly decreased in stroke patients, in both the damaged and healthy hemisphere; (4) The effect changes in FC are a consequence of the stroke damage; (5) The comparison between patients with cortical and subcortical lesions showed significant differences; (6) Stroke effects primarily disrupt whole-brain resting physiology by damaging interregional structural connections rather than only specific gray matter structures.
tumor	19	Aerts et al. (44)	Reduced Wong Wang model	25 tumor patients, 11 healthy controls	T1w, DWI, fMRI	Desikan–Killiany Atlas	TVB	①Global scaling parameter (*G*) of the DMF model, ②the feedback inhibition control parameters (*J_i_*)	(1) Using personalized virtual brain models can significantly improve the prediction accuracy of individual functional connectivity patterns; (2) Local model parameters that can differentiate between regions directly affected by a tumor, regions distant from a tumor, and regions in a healthy brain; (3) Individually optimized model parameters are associated with structural network topology and cognitive performance.
	20	Aerts et al. (45)	Reduced Wong Wang model	*Presurgically*: 25 tumor patients, 11 healthy controls; *postoperatively*: 18 tumor patients, 10 healthy controls	T1w, DWI, fMRI	Desikan–Killiany Atlas	TVB	①Global scaling parameter (*G*), ②inhibitory synaptic weights (*J_i_*)	(1) BNM parameters are relatively stable over time in brain tumor patients who underwent tumor resection, compared with baseline variability in healthy control subjects; (2) The association between global scaling parameter and efficiency of the structural connectivity appears to be particularly robust both before and after surgery.
Schizophrenia	21	Klein et al. (39)	Dynamical mean field (DMF) model	96 healthy participants	sMRI, dMRI, rs-fMRI	Desikan–Killiany Atlas	TVB	①Global coupling (*G*) of brain network connectivity, ②excitatory synaptic coupling (*J_NMDA_*), ③local excitatory recurrence (*w_+_*), ④local feedback inhibitory synaptic coupling (*J_i_*)	(1) NRG1 gene may be related to brain excitatory recurrence or excitatory synaptic coupling potentially resulting in altered E–I balance, thus with potential relevance for psychiatric disorders such as schizophrenia; (2) G/G-carriers (rs3924999) exhibit a significant difference in global coupling and multiple parameters determining E–I balance such as excitatory synaptic coupling, local excitatory recurrence, and inhibitory synaptic coupling.
	22	Mana et al. (40)	Stuart–Landau oscillator	47 schizophrenia patients, 118 healthy controls, 49 bipolar disorder (BD) patients, 39 attention deficit hyperactivity disorder (ADHD) patients	DTI, fMRI	Desikan–Killiany atlas	In-house code	Value of *G* at the optimal model working point	(1) Schizophrenia is characterized by a generalized increase in functional dynamics of the global state; (2) Schizophrenia is characterized by alteration in specific patterns of functional brain organization; (3) It was possible to affect global dynamical patterns to induce a shift toward the pathological condition by shifting the local bifurcation parameter of the network toward positive values; (4) A shift toward the healthy condition can be induced by moving the local bifurcation parameter toward more negative values and therefore reducing rhythmic oscillations (noisy regime).
Parkinson’s disease (PD)	23	Saenger et al. (87)	Supercritical bifurcation model	10 PD patients, 65 healthy controls	T1w, T2w, fMRI	AAL template	In-house code	Intrinsic bifurcation parameters *a* of region models	(1) DBS shifts global brain dynamics of patients toward a healthy regime; (2) DBS helps rebalancing resting-state FC on global aspects of integration and synchronization; (3) Communicability and coherence brain measures during DBS-ON are higher compared to DBS-OFF.

AAL, automated anatomical labelling; Aβ, amyloid beta (Abeta); AD, Alzheimer’s disease; aMCI, amnesic MCI; AN, attention network; BNM, brain network model; DBS, deep brain stimulation; DMF, dynamic mean field; DMN, default mode network; dMRI diffusion MRI; DSI, diffusion spectrum imaging; DTI, diffusion tensor imaging; DWI, diffusion weighted imaging; EEG, electroencephalogram; eLORETA: exact low resolution electromagnetic tomography; EZ, epileptogenic zone; FC, functional connectivity; fMRI, functional MRI; FPN, frontoparietal network; FTD, frontotemporal dementia; HC, healthy control; HCP, Human Connectome Project; LN, limbic network; MCI, mild cognitive impairment; MEG, magnetoencephalogram; MNI, Montreal Neurological Institute; MRI, magnetic resonance imaging; NMM, neural mass model; NRG1, neuregulin-1; PD, Parkinson’s disease; PET, positron emission tomography; PZ, propagation zone; ROI, region of interest; rs-fMRI, resting-state functional MRI; SC, structural connectivity; SCD, subjective cognitive decline; sEEG, stereo EEG; sMRI, structural MRI; SWD, spike-and-wave discharge; T1w, T1 weighted image; T2w, T2 weighted image; TVB, The Virtual Brain; VEP, Virtual Epileptic Patient

### Epilepsy

Epileptic seizures are highly synchronized, high-amplitude pathological neural activities, which are capable of propagating and ceasing throughout cerebral regions through a multitude of varied spatiotemporal patterns.

As mentioned in the “Brain Network Models” section, the Epileptor theoretically and experimentally simulates an exhaustive taxonomy of epileptic events including onset, offset, and the progression traits of seizures [4]. In the past years, the Epileptor and its derivative models have successfully guided translational research on epilepsy, such as characterize spatiotemporal dynamics of seizure spread [33,34], as well as the origins along with the trajectories of spike-wave events (SWEs) throughout ictal episodes [34].

By identifying the EZ and establishing clear benchmarks for assessing the intensity of local epileptogenicity, previous studies delved into the mechanisms of seizure initiation and propagation, offering insights for preoperative assessment of epileptogenicity [5,32]. Using BNM as a tool to explain previously observed diverse phenomena (e.g., seizure initiation, propagation, and termination) of the epileptic brain, Proix [34] investigated and summarized the main mechanism of synchronous termination of seizures that the seizure wavefront is the main cause of seizure activity, and seizures terminate synchronously when the wavefront disappears. For an exception scenario where the SWE source remains stable, they explained the seizure mechanism that one bifurcation of the offset advances more rapidly than the seizure wavefront, encompassing the complete spectrum of engaged neural fields. Furthermore, Courtiol [35] extended the phenomenological Epileptor, enabling it to generate natural brain rhythms as well as the observed epileptiform activity. Their main finding is that in the interictal resting phase, epileptic brain activity is linked to reduced overall excitability due to the working point shift of the model, suggesting that the brains undergoing epilepsy operate closer to a stable equilibrium point than healthy brains. In sum, BNMs enable to investigate the local and global network neurodynamic processes, helping to understand the mechanisms behind the spatiotemporal dynamics of seizure initiation, propagation, and termination. The application of BNMs has potential to help pinpoint seizure onset zones for resection surgery and further guide precise therapeutic plan for individual drug-resistant epilepsy patient.

### AD and PD

AD is a typical neurodegenerative brain disorder that impairs cognition, behaviors, and social skills. Recent connectomics perspectives linked Aβ accumulation or misfolded tau protein propagation to brain functional network reorganization in AD [81]. By characterizing neurophysiological parameters, previous studies suggested that AD pathology can disrupt brain functioning on both local and global level, manifesting as imbalances between the excitatory and inhibitory neuronal populations and alterations in the configuration of global network [82]. For instance, several researches have concentrated on representing alterations within interconnected excitatory and inhibitory neural populations as their correlation to network organization in clinical cohorts [83,84]. Specially, de Haan [84] showed that stimulating selectively on excitatory neurons leads to a prolonged maintenance of oscillation patterns, interconnectivity, and the topology of neural networks. The findings underscore the inherent unpredictability of the impact of excitatory and inhibitory processes on the intricate workings of the brain. Nonetheless, these studies did not establish a direct correlation between these alterations and variations in cognitive functions. Later, Zimmermann [27] elucidated that optimal operational points for excitation, inhibition, and large-scale coupling within a subject-specific model may correspond to variations in cognitive performance across the spectrum from normal aging to conditions such as mild cognitive impairment (MCI) and AD. They modeled the Limbic SubNet and the Whole Network of AD and then found that these 2 models showed opposing brain-behavioral patterns. In the Limbic SubNet, inputs and excitation between brain regions showed an inverse relationship with cognitive abilities, while inhibition demonstrated a direct correlation with cognitive performance. In the Whole Network, excitation was found to have a positive association with cognitive functions, whereas inhibition exhibited a negative relationship with cognition. More recently, Stefanovski [28] proposed to model protein biomarker distribution in a personalized virtual brain using TVB framework. The results demonstrated that individual Aβ loads will affect the excitatory–inhibitory balance in specific brain areas, resulting in heightened neuronal excitability. That is, local Aβ-induced disinhibition and hyperexcitation are posited as potential contributors of AD pathogenesis.

PD is another neurodegenerative disease mainly manifesting movement symptoms, like bradykinesia, resting tremor, and rigidity. The motor dysfunction originates from the loss of dopaminergic neurons among substantia nigra pars compacta [85,86]. DBS serves as an efficacious therapeutic approach for PD, which adepts at controlling the above movement symptoms. However, the intrinsic brain mechanisms after DBS interventions remain largely enigmatic at present, limiting its effect [87]. By applying a supercritical bifurcation NMM, Saenger [87] explored the effects of DBS on the brain in PD, both locally and globally. They showed that the alignment between the fMRI-derived empirical data and simulated data increased rapidly as a function of the coupling strength *G* for all groups. The local dynamics was evaluated with the bifurcation parameter *a* of each brain region. Analyzing the difference of bifurcation parameters between groups, PD patients in the DBS-OFF condition predominantly exhibited negative values of *a*, representing predominantly stable BOLD signals. In contrast, the distribution of patients showed sharper peaks that shifted toward the bifurcation point (*a* = 0) with DBS-ON, similarly to the healthy controls [87]. Notably, the DBS intervention was found to shift the overall brain states of patients in PD toward a healthier state and rebalance the brain abilities of integration, synchronization, and communicability. This evidence indicates that BNM appears to be an effective framework to detect and understand brain dynamics induced by continuous perturbation from the neuromodulation for PD.

### Tumor

In the resection of brain tumors, the objective of presurgical planning is to delineate eloquent cortical areas and white matter tracts near the lesion to avoid damaging them during surgery, thereby preserving crucial brain functions [44]. BNMs have the potential to determine the optimal surgical target by preoperative virtual exploration based on individual patient data [44,45]. Aerts [44] studied brain dynamics prior to tumor resection using personalized BNMs, who found that the optimal local model parameters varied among regions directly impacted by a tumor, regions far from a tumor, and healthy brain regions. They further showed that model parameters optimized individually are linked to both structural network topology and cognitive performance. For example, glioma patients exhibited a noticeable increase in participation coefficient (i.e., the connection strength of a node within its community) compared to healthy controls. Later, they suggested that optimized model parameters remain relatively consistent before and after surgery, in contrast to the baseline variability observed in healthy controls [45]. They also perform a novel “virtual neurosurgery” by BNM, mimicking the actual tumor resection surgery by eliminating white matter fibers within the resection area and simulating neural activity once more. Their experiments showed that existing BNMs have the potential to predict individual brain dynamics following tumor resection surgery, using only preoperatively available information.

### Stroke

The heterogeneity of post-stroke functional recovery continues to pose a significant challenge in the recovery process for stroke patients, likely due to the inherent complexity of damage in a highly interconnected brain. Previous studies have demonstrated that large-scale BNMs are able to track and foresee long-term rehabilitation after stroke [41,42]. Falcon [41] proposed that increasing the parameter governing distant interactions in BNMs (suggesting a preference for regional dynamics over global ones) can be detrimental as it leads to diminished communication due to a decline in operational efficiency. Besides, stroke patients exhibited a uniform decline in conduction velocity and displayed relative increasement in local-over-global brain activities and reduced local inhibitory coupling compared to healthy controls, which revealed a compromised post-stroke system that prefers excitation-over-inhibition and local-over-global dynamics, in accordance with prevailing studies on cerebral ischemic mechanisms within mammalian species [42] . Moreover, parameters ascertained from BNMs were correlated with functional outcomes, indicating that these parameters were able to predict long-term recovery after therapy. As for modeling studies for prenatal or perinatal stroke child patients, Adhikari [58] demonstrated that early injury of brain structural is not likely to cause sustained impact on FC among the affected regions and corresponding areas on the same brain side during resting state. Regions that suffered from early injury may still contribute to the development of quasi-normal capabilities in specific cognitive areas, including linguistic skills, among pediatric populations.

### Schizophrenia

BNM applications can also extend to psychosis. Given correlations between genetic variations within the *NRG1* locus and schizophrenia [88], Klein [39] employed the DMF method to simulate neural activities and study the association between the *NRG1* gene expression levels and neural E–I balance. With multimodal MRI data and DNA samples of totally 96 healthy participants, *NRG1* was observed to impact excitatory and inhibitory neurotransmission, suggesting that BNM is a promising approach to detect disrupted neurobiological pathways in psychiatric disorders.

## Concluding Remarks and Future Perspectives

Recent studies have shown the great potential of BNM in medical applications for various neuropsychological disorders. Given that BNM framework allows to investigate large-scale brain dynamics by linking the simulated functional signals to the empirical neurophysiological data, we believe the aberrant brain dynamics across multiple temporal-spatial scales could be understood theoretically with better interpretations. In this review article, the medical applications of BNMs have fallen into 3 aspects, including (a) exploring neuropathological mechanisms, (b) elucidating therapeutic effects, and (c) predicting disease outcome. For the first aspect, cortical dynamic metrics, such as the degree of E–I balance, can be measured based on the BNM simulation to explain the brain network reconfiguration due to neuropathology [39]. For the therapeutic effects, different local neural oscillatory statuses revealed by a supercritical bifurcation model can specifically guide how neuromodulation like DBS can perturb whole-brain dynamics toward a healthy regime [87]. Last, the estimated local and global coupling coefficients, extracted prior to therapeutic intervention, can improve prediction of individual FC dynamics after tumor resection [44] or post-stroke motor gains [42].

Despite the many advances of the BNMs in medical applications, several limitations should be considered. The estimated model parameters may not be optimized based on single-modality data. For instance, due to the poor temporal resolution of fMRI data, fitting simulated results with empirical data may not be efficient from a computational perspective. Employing the multimodal fusion strategy is advisable for conducting data-based model approaches, which may offer a more comprehensive depiction of brain functional networks than any single modality could achieve. Wei [89] have proposed a multimodal Bayesian fusion rests upon an NMM, which permits every parameter to be informed by insights from MEG/EEG and fMRI empirical data. By Bayesian fusion, EEG inversion is first used to provide a priori distributions (constraints) for neural source parameters as well as to estimate the spatial parameters of the conductor field. Then, fMRI inversion estimates precise neurogenic parameters and region-specific hemodynamic parameters based on the priori distribution derived from EEG. Their research demonstrated that Bayesian fusion serves as an effective strategy, harnessing the distinct temporal resolution of EEG data and the spatial accuracy of fMRI data. Another pitfall lies in that individual SC may not achieve improved forecasts of individual FC configurations. Recent study indicated that subject-specific relationships between SC and FC are restricted, as the disparity among participants in SC is considerably smaller relative to the larger fluctuations observed in FC [90]. At the individual level, a specific structural segmentation of brain networks could lead to distinct manifestations of FC, as the FC dynamics are generated from intricate multisynaptic communications within SC network [48]. Cerebral SC and FC also exhibit disparities in whole-brain organizational patterns. Specifically, within structural connectomes, nodes that share analogous attribute (e.g., degree) have higher probability of interconnection, in contrast to functional networks [48]. Therefore, using NMMs of higher-order interactions to capture personalized FC patterns grounded in SC may result in poor accuracy. In future research, the findings of subject specificity of the SC-FC relationship should be considered. Thus, a BNM formulated from the empirical data of one single subject can be further refined and reliably used in other individual participants. At the group level, the observed low variability underlying the intersubject SC networks might also be due to relatively coarse parcellation schemes (e.g., Desikan–Killiany atlas with 68 parcels [91]) or oversimplified diffusion models (e.g., diffusion tensor MRI and DTI) deployed in the neuroimage preprocessing stage. Therefore, more elaborate parcellations (e.g., Glasser brain atlas with 360 parcels [92] or Julich-Brain atlas [93] with 248 cytoarchitectonic areas), as well as more sophisticated tractograghy techniques (e.g., high angular resolution diffusion imaging or diffusion spectrum MRI [94]), were recommended for improving biological plausibility and individual discriminability of the brain connectome in computational dynamic modeling.

For the future development of the BNM approach in neurological research, attempts to design the next generation of BNMs with finer grained biophysiological details should be encouraged. However, we strongly recommend that medical experts or data scientists give high priority to the choice of model at hand. The choice of the appropriate BNM should reflect the specific research goal and often conforms to the “minimal model approach” [95]. On the one hand, the estimated parameters of each model reflect particular biophysiological properties of interests. For instance, the Jansen–Rit model focuses on firing rates, whereas other models are often employed to estimate field potentials (SJ3D) or phenomenological activities (Epileptor). On the other hand, the choice of the appropriate BNM should also be informed by the neuropathology of specific neurological disorders. For example, where particular neurotransmitter alterations are of interests [e.g., NMDA and GABA (γ-aminobutyric acid) alteration in the basal ganglia–thalamocortical circuit], the DMF model should be considered to assess the potential abnormal synaptic processes of PD patients as a mathematical microscope. When the effects of local oscillatory activities driven by neuropathology (such as regional hyperexcitation caused by high-load Abeta in the AD) are of interests, the Jansen–Rit model is applicable to measure the strength of excitatory and inhibitory connections within the neural population. The Epileptor model should be considered when the pathogenesis source of epilepsy and the effectiveness of treatments are of interests, due to its remarkable performance to monitor the spatiotemporal dynamics of LFP activity throughout the onset, progression, and termination of epileptic events.

Overall, we speculate that the clinical efficacy of neuromodulation treatment for brain diseases would be promoted to a new level in future BNM applications. Recent evidence suggests that control over the synaptic inputs as well as the intrinsic excitability of neurons by manipulating the membrane potentials and ion channels to normalized excitatory level can affect local neuronal responses to DBS [96,97]. Therefore, by determining whether whole-brain functioning of patients rebalance toward a health-aligned state after perturbing local neurodynamic parameters of brain in silico, BNM allows to explore precise sweet spots for therapeutic modulations [87]. Future model development researches for BNM need to estimate more types of neurodynamic parameters (e.g., the inputs of presynaptic signals, frequency-dependent synaptic inhibition, and the inherent excitability within postsynaptic neurons), helping to disclose more detailed neurodynamic process under neuromodulation effects.
